# Supplementary material for: Epigenetic Modulation of TLR4 Expression by Sulforaphane Increases Anti-Inflammatory Capacity in Porcine Monocyte-Derived Dendritic Cells
Source: Biology (Basel). 2021 May 31;10(6):490. doi: 10.3390/biology10060490 (PMC8227201; doi:10.3390/biology10060490)

The raw western blot data for Figure 1B.

The following result were conformed from first independent experiment of duplicate.

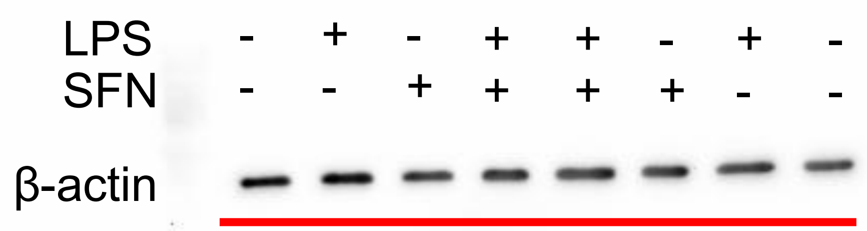

The raw western blot data for Figure 1B.

The following result were conformed from one experiment of duplicate.

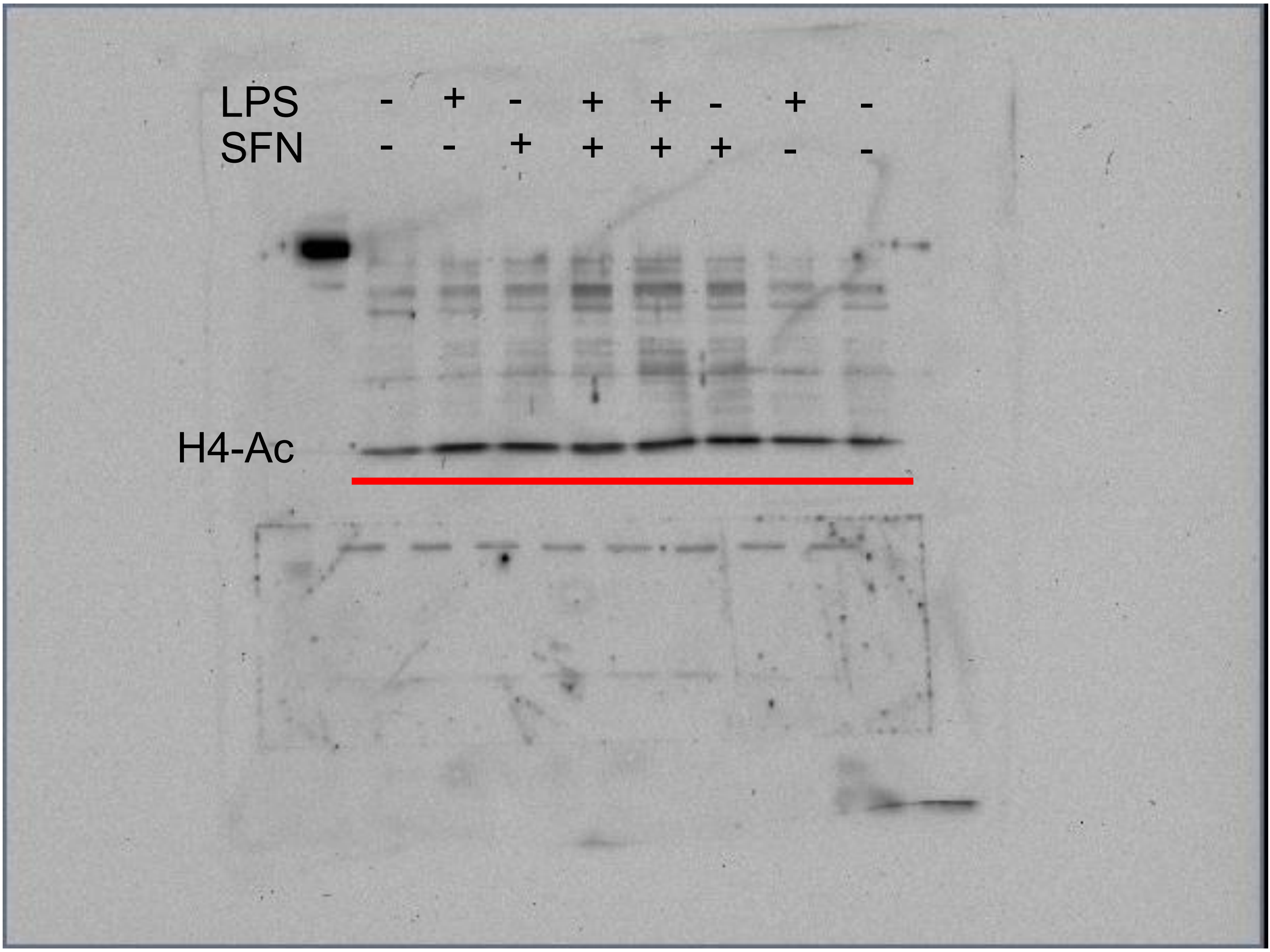

The raw western blot data for Figure 1B.

The following result were conformed from one experiment of duplicate.

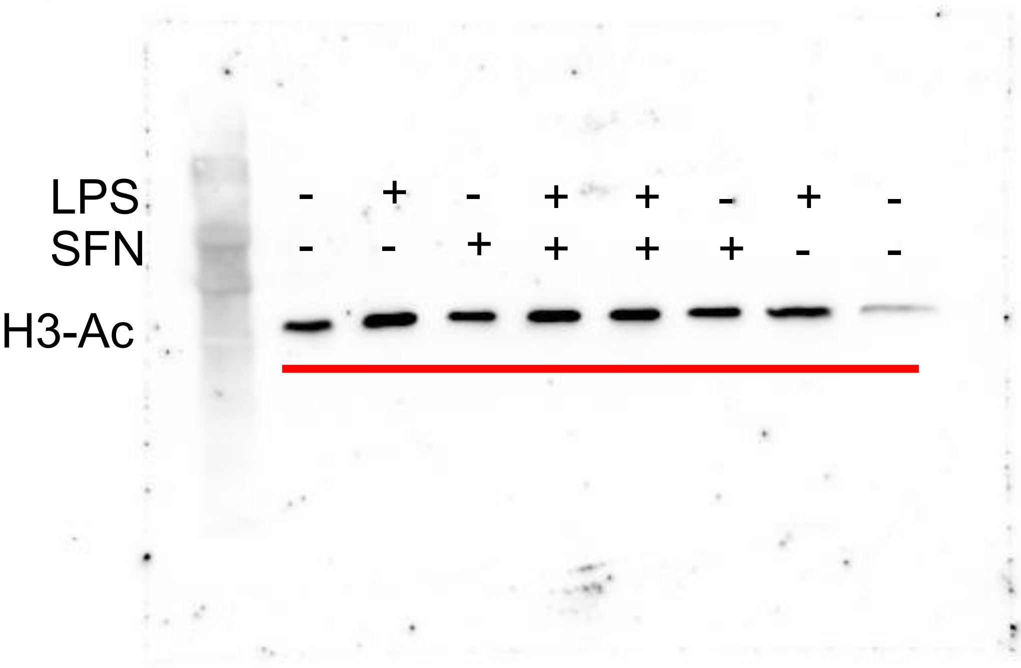

The raw western blot data for Figure 1B.

The following result were conformed from second independent experiment.

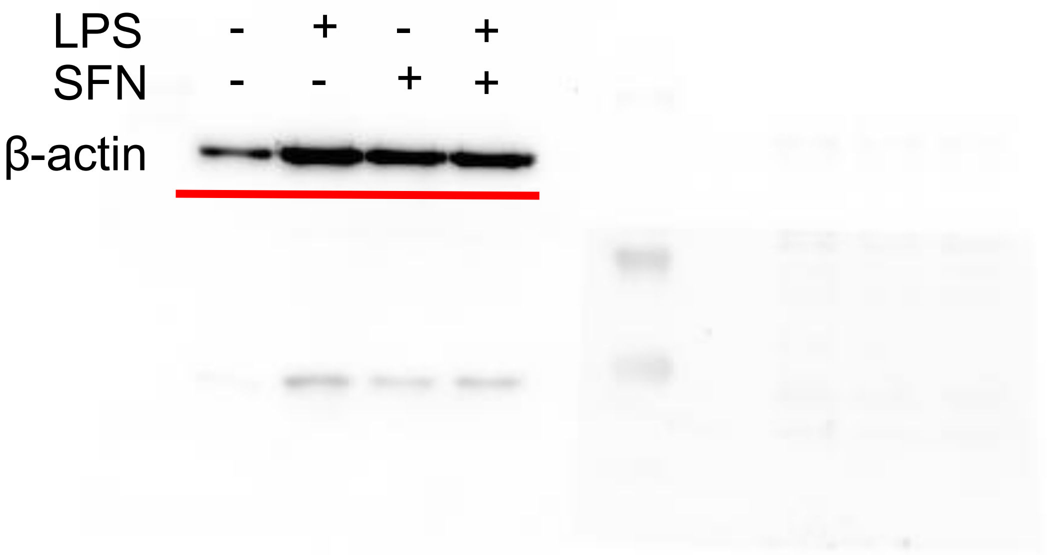

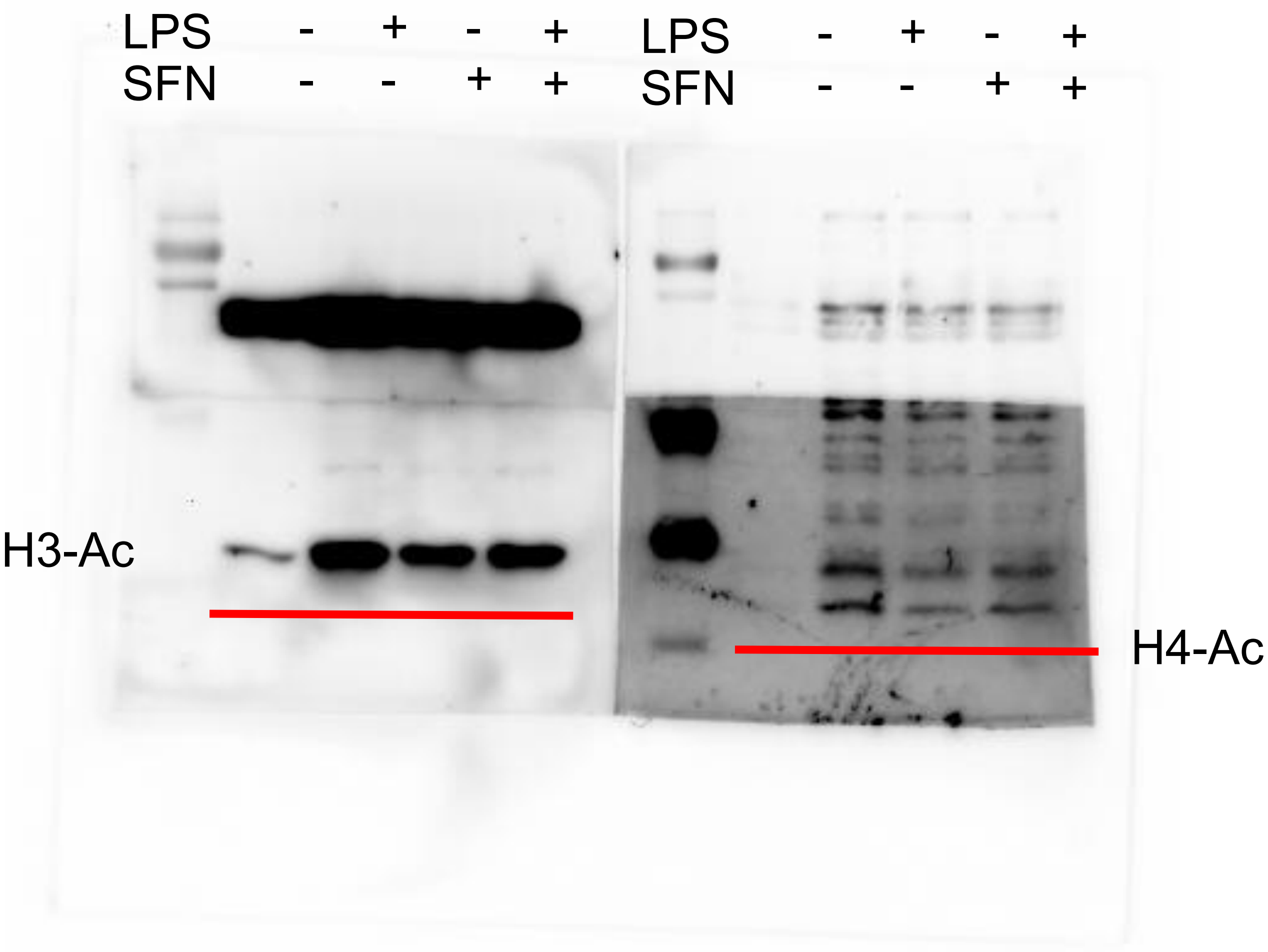

The raw western blot data for Figure 1B.

The following result were conformed from third independent experiment.

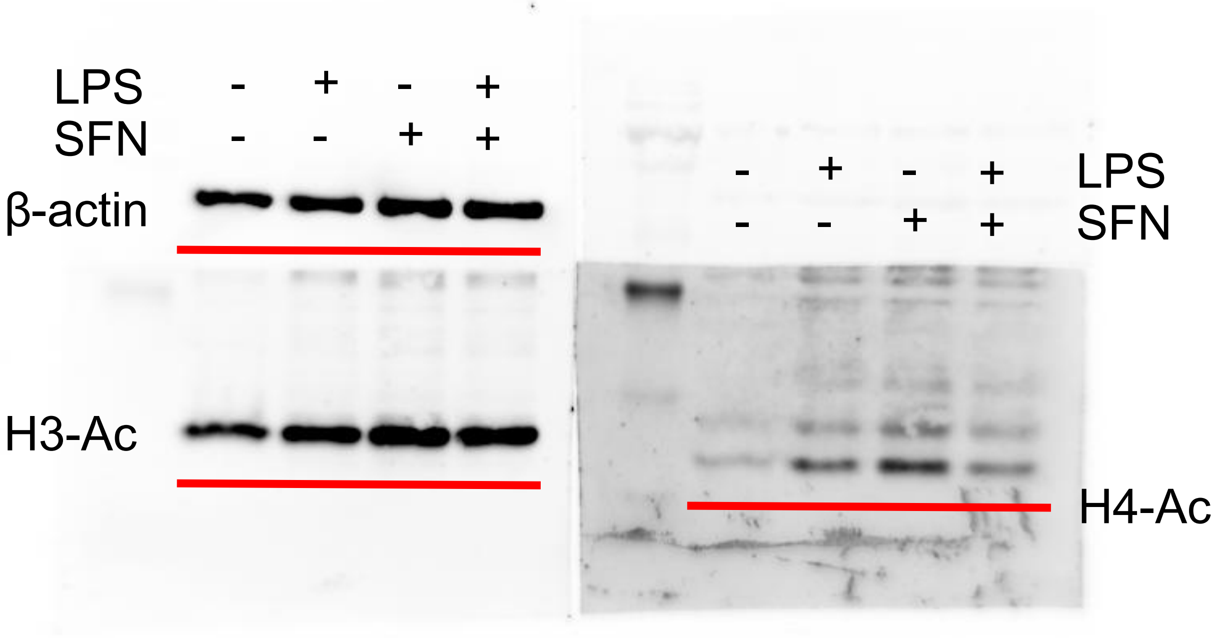

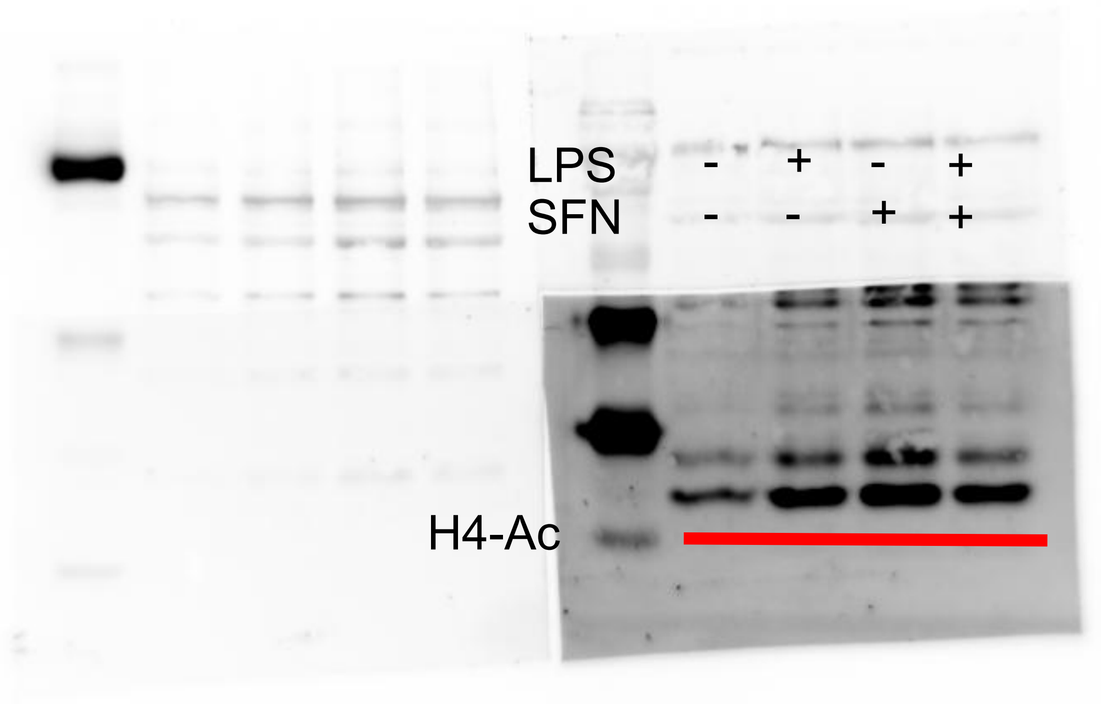

Supplement: Supplementary file 1 [file biology-10-00490-s001.zip › biology-1204387-supplementary.pdf]
